# PI3K/mTOR Pathway Inhibition: Opportunities in Oncology and Rare Genetic Diseases

**DOI:** 10.3390/ijms20225792

**Published:** 2019-11-18

**Authors:** Petra Hillmann, Doriano Fabbro

**Affiliations:** PIQUR Therapeutics, Hochbergerstrasse 60C, 4057 Basel, Switzerland

**Keywords:** PI3K, mTOR inhibitor, cancer, overgrowth syndrome, mTORopathy, TSC, PROS, APDS, PTEN hamartoma, brain penetration

## Abstract

The phosphatidylinositol 3-kinase (PI3K)/mammalian target of rapamycin (mTOR) signaling pathway has been implicated as a cancer target. Big pharma players and small companies have been developing small molecule inhibitors of PI3K and/or mTOR since the 1990s. Although four inhibitors have been approved, many open questions regarding tolerability, patient selection, sensitivity markers, development of resistances, and toxicological challenges still need to be addressed. Besides clear oncological indications, PI3K and mTOR inhibitors have been suggested for treating a plethora of different diseases. In particular, genetically induced PI3K/mTOR pathway activation causes rare disorders, known as overgrowth syndromes, like PTEN (phosphatase and tensin homolog) hamartomas, tuberous sclerosis complex (TSC), phosphatidylinositol-4,5-bisphosphate 3-kinase catalytic subunit alpha (PIK3CA)-related overgrowth spectrum (PROS), and activated PI3-Kinase delta syndrome (PI3KCD, APDS). Some of those disorders likeTSC or hemimegalencephaly, which are one of the PROS disorders, also belong to a group of diseases called mTORopathies. This group of syndromes presents with additional neurological manifestations associated with epilepsy and other neuropsychiatric symptoms induced by neuronal mTOR pathway hyperactivation. While PI3K and mTOR inhibitors have been and still are intensively tested in oncology indications, their use in genetically defined syndromes and mTORopathies appear to be promising avenues for a pharmacological intervention.

## 1. Introduction

Hyperactivation of the phosphatidylinositol 3-kinase (PI3K)/mammalian target of rapamycin (mTOR) signaling pathway has been observed in a high percentage of human cancers inducing growth, survival, and proliferation [1]. PI3K is a lipid kinase that phosphorylates phosphatidyl-inositol-bisphosphate (PIP2) into PIP3. Its function in health and diseases has been the focus of research since the late 1980s [2,3]. PI3K/mTOR signaling components have been prime targets for the development of anti-cancer agents in past years [4,5]. The main targets are the four isoforms of Class I PI3K, PI3K α, β, γ, and δ, as well as the two mTOR complexes known as mTORC1 and mTORC2. Furthermore, inhibitors of downstream targets like protein kinase B (AKT/PKB) are in clinical development for oncology indications. In 2014, the first inhibitor of PI3Kδ, idelalisib (Zydelig^®^, Gilead Sciences, Foster City, CA, USA) was approved for the treatment of chronic lymphocytic leukemia (CLL) followed by three novel drug approvals in 2017, 2018, and 2019. The development and use of PI3K inhibitors in oncology has been reviewed extensively elsewhere [1,6,7,8,9,10,11,12].

**Figure 1:**
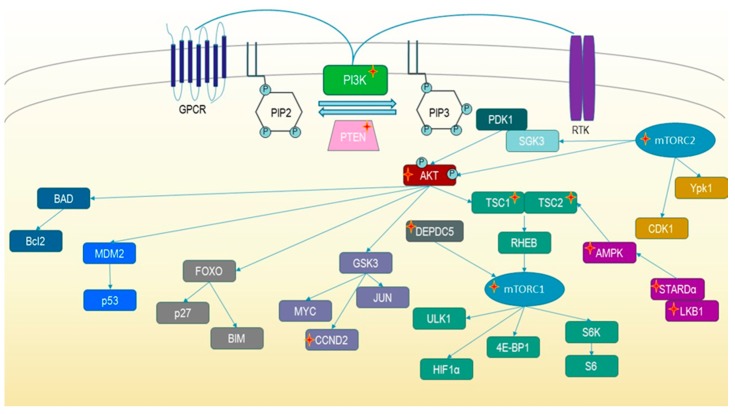
PI3K/mTOR signaling cascade. Arrows address downstream signaling, which leads to inhibition or activation of pathway components resulting in growth, survival, and proliferation. Pathway hyperactivation can be induced by mutations of different genes (marked with a red star) leading to genetic overgrowth syndromes or mTORopathies.

Gain of function (GOF) mutations have been found in Class I PI3K and downstream enzymes like AKT and mTOR. Loss of function (LOF) mutations were detected in PTEN and TSC. All of these mutations lead to an activation of PI3K/mTOR signaling (Figure 1) [1,13,14]. In the catalytic and the regulatory subunits of PI3Kα, PI3Kβ, and PI3Kδ, various GOF mutations have been identified, which lead to diverse human diseases including cancers [4,6,15].

In recent years, sequencing efforts have linked rare genetic disorders like Tuberous Sclerosis Complex (TSC) to hyperactivated PI3K/mTOR signaling. Germline mutations or mosaic mutations of different genes were found in tissue of versatile functions, e.g., in skin, kidney, or brain [15,16,17]. Mutated proteins found in genetic disorders are depicted in Figure 1. The spectrum of genetic PI3K/mTOR pathway associated disorders is broad. Table 1 lists the diseases induced by germline or mosaic mutations of the genes shown in Figure 1 (https://ghr.nlm.nih.gov). Many indications are associated with mostly benign tumors or present with overgrowth of organs and body parts including the central nervous system (CNS) [14,18,19,20]. Onset is often observed in childhood and a lifelong suppression of the pathway may diminish or prevent disease symptoms. Currently available therapies—if any—include the rapalogs rapamycin or everolimus, which are, although exquisitely selective, immune suppressive. Most of the catalytic PI3K and mTOR inhibitors that are used in the clinic are not optimized for treating indications that present with CNS symptoms since their pharmacokinetic (PK) properties prohibit brain penetration.

## 2. Clinical Development of PI3K and mTOR Inhibitors in Oncology

### 2.1. Clinical Stage Compounds

**Figure 2:**
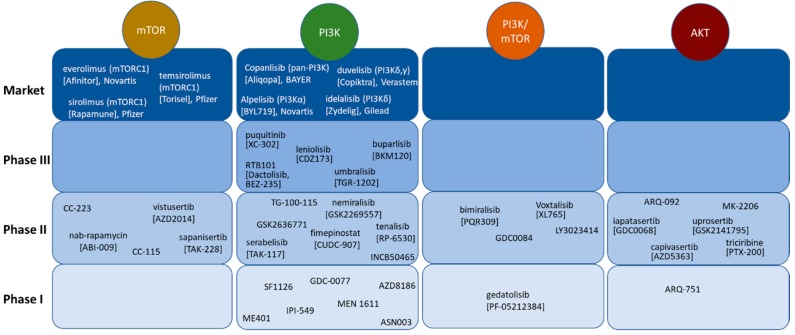
mTOR inhibitors, PI3K inhibitors, dual PI3K/mTOR inhibitors, and AKT inhibitors in clinical development. Drugs are sorted by the stage of their clinical development (Phase I to market) as well as by their mode of action inhibiting AKT, mTOR, PI3K, or PI3K and mTOR. Among PI3K targeting agents, pan-PI3K inhibitors and isoform specific compounds are in clinical development.

Large pharma players and small biotech companies have invested in the development of inhibitors of PI3K signaling to target cancers. PI3K and mTOR inhibitors as well as inhibitors of AKT that are currently in clinical development are depicted in Figure 2 and are deposited in https://www.guidetopharmacology.org/targets.jsp.

While most compounds bind to the ATP-pocket of PI3K and/or mTOR, rapamycin and its analogs are allosteric inhibitors of mTORC1 targeting protein-substrate phosphorylation (mainly S6K) via FK506 binding protein (FKBP) association [21]. Three allosteric inhibitors of mTORC1, the so called rapalogs sirolimus, everolimus, and temsirolimus, have been approved not only in oncology but also as immune suppressants and for neurological disorders. Everolimus (Novartis, Basel, Switzerland), for example, is on the market under three trade names: Afinitor^®^ for the treatment of breast cancer, neuroendocrine tumors, and renal cell cancer, Zortess^®^ (USA)/Certican^®^ (Europe) in combination with cyclosporin to prevent rejection of organ transplants, and Votubia^®^ for TSC-associated tumors and refractory epilepsy. In addition, an everolimus-eluting stent has been approved for treating de novo coronary artery lesions (Promus^®^, Boston Scientific, Marlborough, MA, USA). In contrast, the ATP-competitive TOR kinase inhibitors (TORKi), which inhibit both mTOR complexes, mTORC1, and mTORC2, are in earlier stages of clinical development (Figure 2). CC-223 (Celgene, Summit, NJ, USA), CC-115 (dual DNA-PK/mTOR inhibitor, Celgene), vistusertib (Astra Zeneca, Cambridge, UK), and sapanisertib (Takeda, Tokyo, Japan) are in Phase II in oncology indications (www.clinicaltrails.gov) [22,23,24,25,26].

PI3K inhibitors are sub-divided in inhibitors targeting one or two isoforms only and compounds that are pan-PI3K inhibitors, which are potently binding to all PI3K isoforms. Inhibitors of PI3Kγ and δ like duvelisib (Verastem, Needham, MA, USA) and idelalisib (Gilead, Foster City, CA, USA) are limited to administration in patients with cancers of the immune system since these two PI3K isoforms are expressed in immune cells only [27,28]. The development of dual PI3K/mTOR kinase inhibitors is still in an earlier stage. Bimiralisib (Piqur, Basel, Switzerland), GDC0084 (Kazia, Barangaroo, Australia), and LY3023414 (Ely Lilly, Indianapolis, IN, USA) are in Phase 2 clinical development in oncology single agent and in combination (Figure 2). Dactolisib (BEZ235, RTB-101(ResTORbio, Boston, MA, USA)) has been evaluated in oncology and is also undergoing clinical testing in respiratory illness [29,30].

A range of AKT inhibitors is in clinical development for various oncology indications. While most compounds bind to the ATP site of AKT, ARQ-092 (ArQule, Burlington, MA, USA), MK-2206 (Merck Co., Kenilworth, NJ, USA), and ipatasertib (Roche, Basel, Switzerland) are allosteric inhibitors of AKT. ARQ-751 (ArQule) is in Phase I clinical development. ARQ-92, MK2206, ipatasertib as well as capivasertib (Astra Zeneca), uprosertib (GSK, Brentford, UK), and triciribine (Prescient Therapeutics, South Melbourne, Australia) have made it into Phase II trials in oncology indications (Figure 2). ARQ-092 is tested in patients with PROS and Proteus Syndrome (NCT03094832).

Besides the compounds listed in Figure 2, efforts are ongoing to target other components of the PI3K signaling cascade like S6 kinase (S6K), phosphatidylinositol-4-phosphate 3-kinase C2 domain-containing alpha polypeptide (PIK3C2), and Unc-51-like autophagy activating kinase (ULK) [31,32].

Most compounds are currently being evaluated in solid and hematological cancers. Some molecules that cross the blood-brain barrier are being tested in cancers of the CNS like glioma, glioblastoma, or in brain metastases [33,34,35].

### 2.2. Sensitivity Markers and Patient Selection

Even after years of PI3K inhibitor development, no clear sensitivity markers have been found that would predict better responses in patients. Many screens of cancer cell lines were performed. While some cell populations showed a higher degree of sensitivity for cell lines carrying PI3K mutations, other populations did not indicate a clear correlation. Negative predictors have been identified on an experimental level. Activation of the MAPK pathway via B-Raf proto-oncogene (BRAF) or RAS (RAS GTPase) mutations are associated with resistance to PI3K inhibition. These mutations might be used as negative predictors for drug efficacy [36,37,38,39,40,41]. However, exclusion of patients with tumors that carry mutations predicting treatment resistance was never translated into clinical testing. Many clinical trials have been performed with patients that were not carefully selected for a response. More recently, pre-selection of particular patient groups has become mandatory. For example, LY3023414 is tested in a Phase 2 study in children with solid tumors, non-Hodgkin’s lymphoma or histiocytic disorders with TSC or PI3K/mTOR mutations and bimiralisib is under clinical investigation in head and neck squamous cell carcinoma harboring a NOTCH1 loss of function mutation [42,43]. The recent approval of alpelisib (Piqray^®^, Novartis, Basel, Switzerland) for treating hormone responsive breast cancer with GOF mutations in PIK3CA shows that patient selection for particular mutations, although not perfect, may be beneficial. Patients without PI3K mutation had no benefit in progression-free survival while patients with mutations clearly responded when treated with alpelisib in the Phase 3 SOLAR-1 trial [44].

### 2.3. Tolerability in the Oncology Setting 

Systemic toxicity has been a major hurdle for the development of PI3K inhibitors, which becomes even more prominent when discussing the treatment of non-oncology indications. To achieve sufficient efficacy, about 90% of signaling inhibition in the tumor appears to be required. This is occasionally only achievable at doses that are associated with adverse events [1]. This may, in part, be due to the fact that GOF mutations in the PI3K/mTOR pathway do not appear to be strong drivers of cancer growth.

The spectrum of adverse events for rapalogs, TORKi, and PI3K inhibitors varies and the development of many compounds has been stopped due to unacceptable side effects. All approved molecules have warnings on their label regarding immune suppression including risk of infections or pneumonitis. Dose-limiting toxicity derived by immune-suppressive action is particularly prominent for rapalogs. Pneumonitis, stomatitis, and infections are commonly observed [45,46,47]. Rapalogs have numerous effects on components of the immune system, as reviewed extensively elsewhere [1,48,49,50]. In addition, PI3K inhibitors induce hyperglycemia (particularly when inhibiting the PI3K alpha isoform), rash, and fatigue. Among adverse events reported for buparlisib (BKM120, Novartis, Basel, Switzerland), a brain penetrant pan-PI3K inhibitor, was a high rate of depression [51,52,53]. Since buparlisib appears to have an off-target liability with respect to inhibition of microtubule polymerization, the depression might be related to its disruption of tubulin polymerization in the CNS rather than to on-target activity on PI3K [54]. There is only limited data available for clinical testing of TORKi. AZD2014 and CC-223 showed reduced immune suppression compared to rapalogs and were relatively well tolerated in first oncology trials [23,55].

Most clinical trials with PI3K/mTOR pathway inhibitors have employed daily dosing regimens. In many patients, dose reduction or treatment interruption was necessary. Scheduling schemes provide an option to reduce toxicity. In animal models, intermittent dosing led to similar or even increased efficacy accompanied by a higher level of tumor cells entering apoptosis as observed for copanlisib, AZD8835, and the TORKi AZD2014 [56,57,58]. Intermittent dosing schedules are also evaluated in the clinic, e.g., for copanlisib, bimiralisib, and serabelisib, with the goal to increase target inhibition while decreasing systemic toxicity. Combination of PI3K or mTOR inhibitors with chemotherapy or other targeted agents can be particularly challenging since combination partners often induce similar adverse events.

The therapeutic window for genetic disorders induced by mutations of the PI3K signaling pathway like TSC or PROS might be larger. In this case, it is not clear to what extent the pathway needs to be shut down to reach efficacy, but potentially bringing PI3K signaling back to normal may be sufficient. On the other hand, dose scheduling has not been evaluated in these disorders and it is possible that continuous pathway inhibition is the best way forward.

## 3. Clinical Development of PI3K and mTOR Inhibitors in Non-Oncological Indications

The development of PI3K and mTOR inhibitors has concentrated on oncology indications, but some promising efforts have been taken to further broaden the scope of those molecules to benign overgrowth.

Historically, a broad range of conditions characterized by benign overgrowth of the whole body or body parts were named overgrowth syndromes. Overgrowth can often be observed in the fetus and patients affected have an increased risk for developing cancer. Genetic overgrowth syndromes are a subgroup that is caused by germline or somatic mutations of the PI3K/mTOR signaling pathway (Figure 1). In patients affected, generalized or segmental overgrowth can be observed sometimes. This is accompanied by visceromegaly, macrocephaly, or neurological symptoms like autism and learning deficits [17]. There is some overlap between overgrowth syndromes and mTORopathies. mTORopathies are genetic disorders, which are induced by neuronal mutations in the mTOR signaling cascade that lead to hyperactivation of the pathway. They are characterized by treatment resistant epilepsy and other neurological alterations. Conditions like TSC can present with overgrowth as well as CNS manifestations.

### 3.1. PTENopathies or PTEN Hamartoma Tumor Syndrome

The Phosphatase and Tensin Homolog PTEN is a tumor suppressor and negative regulator of PI3K function catalyzing the reverse reaction, the dephosphorylation of PIP3 to PIP2. PTEN loss-of-function mutations are prominent PI3K signaling activators in many tumors [59,60,61] (Figure 1). In 1997, Liaw et al. described germline mutations of PTEN in families with Cowden Disease and Lhermitte-Duclos disease [62]. Today, Cowden Syndrome (CS), Lhermitee-Duclos disease, and Bannayan-Riley-Ruvalcaba-Syndrom (BRRS) belong to the so-called PTENopathies or PTEN hamartoma tumor syndromes, which are a spectrum of diseases induced by PTEN loss-of-function mutations.

While patients with CS develop multiple benign or malignant hamartomas in different organs like thyroid gland, breast, endometrium, and skin and often have a macrocephalus, patients with BRRS mostly suffer from macrocephalus accompanied by hemangiomas, lipomas, a delay of motoric development, and mental retardation [17,63]. There is no therapy for PTENopathies at the moment. Patients undergo constant cancer screening and surgery to remove painful or malignant lesions. Sirolimus has been tested in patients with Cowden Syndrome (NCT00971789). Everolimus and two PI3K inhibitors, dactolisib and BGT226, are under investigation in patients with CS or PTEN hamartoma (NCT02991807, NCT00620594, NCT00600275). Some benefit has been reported for sirolimus treatment. Since patient cohorts are small, sufficient clinical data is missing to evaluate the success of the approach [64,65].

### 3.2. PI3K Related Overgrowth Syndrome (PROS)

PI3K related Overgrowth Syndrome (PROS) comprises several pathological conditions, which present with segmental overgrowth. The same hotspot mutations found in cancers are also present in PROS, mostly in the mosaic state: H1047R in the kinase domain as well as E542K and E545K in the helical domain of the enzyme. In some cases, mutations of AKT1 as well as PTEN have been described, and more mutational data is being collected using improved sequencing technology and availability. Despite the high sensitivity of next-generation-sequencing approaches, low a mutant allele level/degree of mosaicism (sometimes <1%) is still hard or impossible to be detected in patients’ blood. Therefore, overgrown tissue needs to be used for sequencing [17,66,67,68].

The following segmental overgrowth syndromes are grouped under the term PROS: congenital lipomatous overgrowth with vascular, epidermal, and skeletal anomalies (CLOVES), megalencephaly capillary malformation (MCAP), Klippel-Trenaunay syndrome, fibroadipose hyperplasia, Proteus Syndrome, hypoinsulinaemic hypoglycaemia with hemihypertrophy, and other extremely rare disorders [17]. PROS are associated with an increased risk of Wilms tumors, but the prevalence seems low and has not been accurately assessed [69].

Clinically, the spectrum of the disorders correlates with the mutation and the degree of mosaicism. The severity is highly variable and ranges from localized overgrowth of a digit to life-threatening overgrowth affecting vessels or critical organs. Manifestation starts at birth and a range of tissues can be affected including vasculature, lipid tissue, bone, brain, peripheral nerves, liver, and cardiac muscle [19,70].

Currently, there is no approved targeted therapy for PROS. Patients undergo surgical debulking and blocking of overgrowth vessels [19]. A limited number of clinical reports has shown proof of concept for inhibitors of the PI3K/mTOR pathway. For example, single case studies of the use of rapamycin or AKT inhibitors in proteus syndrome are available [65,71]. PI3K inhibitors can potentially be used as single agent at a low dose. Alpelisib (BYL719, Piqray^®^) was tested in 19 patients with PROS at low doses and improved disease symptoms including vascular tumors, hemihypertrophy, and scoliosis [72]. The AKT inhibitor ARQ-092 led to the remission of a cancer in a patient with Proteus Syndrome carrying an AKT1 mutation, and is currently undergoing clinical evaluation in PROS [73].

### 3.3. APDS: Activated Phosphoinositide 3-Kinase δ Syndrome

APDS, which is also known as PASLI disease (p110 δ-activating mutation causing senescent T cells, lymphadenopathy, and immunodeficiency), are combined immunodeficiencies resulting from GOF mutations in the genes *PIK3CD* and *PIK3R1* encoding the subunits of phosphoinositide 3-kinase δ (PI3Kδ) and p85α, the regulatory subunit of PI3K [74,75]. Depending on which subunit is mutated, PIK3CD or PIK3R1, there are two types of APDS termed APDS1 and APDS2, respectively. Both result in hyperactivation of the PI3K/AKT/mTOR/S6K signaling pathway. Patients with APDS may develop immunodeficient and immunodysregulatory features including recurrent respiratory tract infections, bronchiectasis, herpesvirus infections, autoimmunity, non-neoplastic lymphoproliferation, and lymphoma, as well as neurodevelopmental delay and growth retardation [4,5]. In vitro and in vivo effects of inhibiting PI3Kδ by APDS with leniolisib (CDZ173), which is a selective PI3Kδ inhibitor, caused dose-dependent suppression of PI3Kδ pathway hyperactivation [76]. A clinical trial with oral leniolisib in patients with APDS as well as with Sjoberg diseases led to improve immune regulation and to a dose-dependent reduction in PI3K/AKT pathway activity [77].

### 3.4. mTORopathies

Besides its role in metabolism and survival, mTOR has critical functions in brain-specific mechanisms such as synaptic plasticity, learning, and cortical development. The role of mTORC1 in neurosciences and growth has been described well while the role of mTORC2 is still subject to discussion [78].

mTORopathies are rare genetic disorders that are induced by neuronal mutations in the mTOR signaling cascade that lead to hyperactivation of the pathway. They present with mostly treatment-resistant epileptic seizures. Targeted therapies with catalytic mTOR inhibitors may inhibit seizures, and positively influence the progression of the disease (epileptogenesis) as well as other symptoms like behavioral changes, and learning deficits. Among mTORopathies are diseases that also have a broad spectrum of manifestations including overgrowth of the brain, such as in hemimegalencephaly or benign tumors, as observed in Tuberous Sclerosis Complex (TSC). It is unknown how activated neuronal mTOR signaling induces epileptic seizures on a cellular or molecular level [79,80,81].

#### 3.4.1. TSC

Tuberous Sclerosis Complex (TSC) comprises a spectrum of diseases ranging from tumor growth in the brain and in other vital organs (Table 2) to epileptic seizures, behavioral changes, autism, and other TSC-associated neuropsychiatric disorders (TAND). One in 6000 newborns is affected. mTOR signaling is upregulated via germline or mosaic mutation of the TSC1 or TSC2 gene (Figure 1). One third of TSC cases are autosomal dominant inherited while the other two-thirds of cases occur spontaneously.

At present, there is a high medical need since therapy options are sparse. While inhibition of mTOR signaling by rapalogs has positive effects on behavior, reduces tumor formation, and suppresses seizures in animal models [82,83], their chronic use is hampered by their immunosuppressive nature. Everolimus, which originally has been used as an immune suppressant for organ transplants (Certican^®^/Zortress^®^, Novartis, Basel, Switzerland), has been approved for TSC oncology manifestations like renal angiomyolipoma and subependymal giant cell astrocytoma (SEGA) as Afinitor^®^. For treating angiofibroma, the disfiguring skin manifestation of TSC, topical rapamycin is under clinical evaluation and has been approved in Japan only as Rapalimus gel^®^ (Nobelpharma, Tokyo, Japan) [84,85].

In addition, 90% of TSC patients develop epilepsy, which mostly starts under the age of three. For TSC epilepsy, standard anti-seizure drugs (ASD) like levetiracetam or vigabatrin are often ineffective and only treat the disease symptomatically at best [78,86]. Everolimus (as Votubia^®^) has recently also been approved for ASD-refractory partial-onset seizures in TSC patients [46,87,88]. Systemic exposure of rapalogs are, however, known to lead to immune suppression requiring dose reduction in the clinic [46,89]. TORKi may overcome these issues and have shown preclinical proof of concept [90]. Current clinical stage compounds lack sufficient penetration over the blood-brain barrier and, therefore, are not under development for TSC epilepsy, but preclinical candidates with an improved PK profile may be able to fill this gap [90,91]. TORKi clinical development in the oncology field implies a better safety profile compared to rapalogs and, therefore, catalytic mTOR kinase inhibitors may be suitable for TSC oncology indications.

#### 3.4.2. Focal Cortical Malformations (FCMs)

Focal cortical malformations (FCMs) are localized structural defects in the CNS associated with severe epilepsies, which mostly occur in infancy or childhood. Besides seizures, patients suffer from impaired cognition and behavior at different degrees [18]. Recent sequencing efforts in the epilepsy field have linked FCMs to genes regulating the mTOR pathway and, consequently, to local hyperactivation of PI3K/mTOR signaling in the brain. Therefore, mTOR inhibitors have emerged as a treatment option for affected patients [16]. Additionally, focal cortical dysplasia (FCD), hemimegalencephaly, Pretzel syndrome, megalencephaly capillary malformation syndrome, familial focal epilepsy with variable foci, and megalencephaly-polymicrogyria-polydactyly-hydrocephalus syndrome are linked to the mTOR pathway via mosaic mutations of various genes including hamartin/tuberin, mTOR, PTEN, CCND2, DEPDC5, STRADα, AMPK, NPRL3, P85β, or PI3K (Figure 1) and, thus, fall into the category of mTORopathies [18,79,94,95,96,97,98,99]. On the other hand, many of the FCMs come with hyperproliferation of the brain and, therefore, hemimegalencephaly can be listed as an overgrowth syndrome, too.

The prevalence of these rare diseases is often unknown, since no sequencing effort has been previously undertaken to identify related mutations (https://ghr.nlm.nih.gov). Numbers of patients appear far bigger than originally predicted considering that 5% to 25% of focal epilepsies, which is a group of epilepsies that sums up to around 60% of all seizure types, belong to the group of FCD, and at least 30% of those cases show mutations in the mTOR pathway (www.epilepsy.com) [79,100,101]. Sequencing initiatives are ongoing and will further enlighten the role of the mTOR pathway in FCMs.

So far, treatment is limited to conventional, often ineffective anti-seizure medication, corticosteroids and risky epilepsy surgery, leaving a high percentage of patients with untreatable disease progression. Animal models that represent focal and genetic background of the diseases are slowly developing and could help show proof of concept for novel drugs [95,102]. Everolimus is currently undergoing clinical trials in FCD (NCT03198949 and NCT02451696) facing the same drawbacks described for TSC. No further clinical trials in FCMs have been listed for TORKi or for other pathway inhibitors.

### 3.5. Advanced Therapies for Genetic Overgrowth Syndromes and mTORopathies

Although still in an experimental phase, first attempts have been made to test advanced therapies like targeting microRNAs (miRNA) or viral gene therapy to alter PI3K/mTOR signaling. For example, inhibition of miRNA-21-5p induced inhibition of PI3K signaling and a decrease in tumor cell growth in animal models [103]. Virus-mediated hamartin gene therapy led to an improved phenotype in TSC mice [104], and mTOR has been targeted by siRNA nasally to influence aspergillosis in a mouse model. mTOR antisense oligonucleotides were shown to decrease polycystic kidney disease in mice [105,106]. Delivery of RNA or viral drugs to the brain via intrathecal injection is clinically feasible for genetic disorders, as shown for an antisense oligonucleotide tested in patients with spinal muscular atrophy, and also novel delivery systems like nanoparticles will help to target the CNS [107]. More attempts towards gene silencing or gene editing can be expected in the area of mTORopathies [108].

## 4. Conclusions

Thirty years after the discovery of PI3K, the first PI3K inhibitors are on the market for oncology indications. Rapalogs are approved for indications not only in the oncology space but also as immune suppressants as well as for use in rare genetic tumors and epilepsy associated with TSC. While genetic disorders comprising overgrowth and neurological symptoms give a clear rationale for the application of PI3K/mTOR inhibitors in oncology, the prediction of sensitivity or resistance is still subject to discussion and evaluation. Many compounds have failed during development due to unacceptable toxicity. Altered treatment schemes like dose scheduling as well as novel compounds that display enhanced isoform selectivity may increase the therapeutic window in tumor patients.

Clinical data indicating the efficacy of PI3K or mTOR inhibitors in rare genetic disorders associated with constitutive PI3K pathway activation is sparse but promising. For the development of compounds in disorders like PROS and FCD, additional hurdles concerning acceptable tolerability for the use in children and for a lifelong therapy option along with penetration over the blood-brain-barrier need to be taken. For PROS, PTEN hamartoma, and mTORopathies, it can be anticipated that efficacious doses may be lower than in cancer therapy. Bringing the pathway “back to normal” instead of completely shutting it down may be sufficient, as exemplified by the use of alpelisib in PROS. Following this assumption, a higher degree of tolerability and a larger therapeutic window can be expected for the use of kinase inhibitors in this context. Further evaluation of this hypothesis is needed in additional genetic disorders preclinically and in the clinical setting. The use of rapalogs in TSC and overgrowth syndromes is limited by its immune suppressive adverse events. First clinical data from TORKi give hope that this type of compounds can overcome the drawbacks of rapalogs including immune suppression and feedback activation. Compounds with improved brain penetration are needed for the use in mTORopathies and formulation efforts that use nanoparticles or simple prodrug strategies may help address CNS manifestations.

## Figures and Tables

**Table 1:** Gene mutations of the PI3K/mTOR signaling pathway and associated genetic syndromes.

Mutation	Disease
PIK3CA	PROS: Megalencephaly-capillary malformation syndrome, CLOVES syndrome, hemimegalencephaly, fobroadipose hyperplasia, congenital lipomatous overgrowth, Klippel-Trenaunay syndrome
PIK3CD	Activated phosphoinositide 3-kinase δ syndrome (APDS)
PIK3R1	APDS
PTEN	PTEN hamartoma tumor syndrome: Bannayan-Riley-Ruvalcaba syndrome, Cowden syndrome
mTOR	Smith-Kingsmore syndrome, focal cortical dysplasia, hemimegalencephaly
AKT	Proteus syndrome, megalencephaly-polymicrogyria-polydactyly-hydrocephalus, megalencephaly
TSC1/2	Tuberous Sclerosis Complex, focal cortical dysplasia
AMPK	Focal cortical malformation
DEPDC5	Familial focal epilepsy with variable foci, familial mesial temporal lobe epilepsy
CCND2	Megalencephaly-polymicrogyria-polydactyly-hydrocephalus
STRADα	Polyhydramnios, megalencephaly, symptomatic epilepsy, Pretzel syndrome
LKB1	Peutz-Jeghers syndrome

**Table 2:** Manifestations of TSC. TSC is a spectrum disorder that presents with various symptoms in each single patient [92,93]. The table lists the incidence of each manifestation.

Organ	Manifestation	Incidence in TSC Patients
Brain	Epilepsy	90%
	SEGA	10–15%
	Autism	40%
	TAND	90%
Heart	Cardiac rhabdomyoma	90% in infants 20% in adults
Eyes	Retinal hamartoma	50%
Kidney	Angiomyolipoma	70%
	Cysts	35%
	Renal cell carcinoma	2–3%
Lung	Lymphangioleiomyomatosis (LAM)	30–40% of women
Skin	Angiofibroma	75%
	Ungual fibroma	80%
	Fibrous cephalic plaques	25%
	Shagreen patches	50%

## Abbreviations

ASD	Anti-seizure drug
AKT	Protein kinase B (PKB)
APDS	Activated phosphoinositide 3-kinase δ syndrome
BRRS	Bannayan-Riley-Ruvalcaba-Syndrom
CLL	Chronic lymphatic leukemia
CLOVES	congenital lipomatous overgrowth with vascular, epidermal, and skeletal anomalies
CNS	Central nervous system
CS	Cowden Syndrome
FCD	Focal Cortical Dysplasia
FCM	Focal cortical malformation
GOF	Gain of Function
GPCR	G protein-coupled receptor
LOF	Loss of Function
MCAP	megalencephaly capillary malformation
mTOR	Mammalian target of rapamycin
PI3K	Phosphatidyl inositol-3-kinase
PIP2	Phosphatidylinositolbisphosphate
PK	Pharmacokinetic
PROS	PIK3CA-related overgrowth spectrum
PTEN	Phosphatase And Tensin Homolog
RTK	Receptor tyrosine kinase
SEGA	Subependymal giant cell astrocytoma
TAND	TSC-associated neuropsychiatric disorders
TORKi	mTOR kinase inhibitor
TSC	Tuberous Sclerosis Complex
